# High content reduced graphene oxide reinforced copper with a bioinspired nano-laminated structure and large recoverable deformation ability

**DOI:** 10.1038/srep33801

**Published:** 2016-09-20

**Authors:** Ding-Bang Xiong, Mu Cao, Qiang Guo, Zhanqiu Tan, Genlian Fan, Zhiqiang Li, Di Zhang

**Affiliations:** 1 State Key Laboratory of Metal Matrix Composites, Shanghai Jiao Tong University, 800 Dongchuan Road, Shanghai, 200240, China

## Abstract

By using CuO/graphene-oxide/CuO sandwich-like nanosheets as the building blocks, bulk nacre-inspired copper matrix nano-laminated composite reinforced by molecular-level dispersed and ordered reduced graphene oxide (rGO) with content as high as ∼45 vol% was fabricated via a combined process of assembly, reduction and consolidation. Thanks to nanoconfinement effect, reinforcing effect, as well as architecture effect, the nanocomposite shows increased specific strength and at least one order of magnitude greater recoverable deformation ability as compared with monolithic Cu matrix.

Due to the noteworthy mechanical and functional properties exhibited by natural biological materials, bioinspired materials and design have attracted considerable attention^1^,^2^,^3^,^4^. Biological materials are natural composites usually consisting of non-mineralized (“soft”) and mineralized (“hard”) parts, which could provide valuable guidance for design on constituents and architectures in fabricating advanced artificial composites. Nano-laminated structure is widely observed in biological materials, such as nacre, sponge spicule, arthropod cuticle, bone and tooth enamel^5^,^6^. They exhibit some unique features in common: *e.g.* strengthening effect provided by high content of “hard” mineral crystals, their alignment that orient to maximize performance for required loading conditions, and toughening effect conferred mainly by crack passivation and deflection at inter-lamella interfaces^7^. These constituent-structure-property relationships have inspired a large class of bioinspired advanced materials mainly in polymer and ceramic systems^8^,^9^,^10^,^11^,^12^,^13^,^14^,^15^.

Graphene is a single-atomic material with outstanding mechanical, electrical and thermal properties^16^. In last decade, there has been growing interest and progress in the design and fabrication of graphene macrostructures, such as aerogel^17^,^18^,^19^,^20^, fibres^21^ and papers^22^, for effective utilization of their remarkable properties at the macroscale. Meanwhile, fabricating graphene reinforced composites is another important approach for transferring its excellent properties at the nanoscale to the macroscale^23^,^24^,^25^. Moreover, its intrinsic two-dimensional geometry makes graphene an ideal candidate for fabricating nano-laminated composites. To date, most work in the area of graphene reinforced composites has focused on property modification using only small amounts of randomly dispersed graphene^26^,^27^,^28^. This is in stark contrast to natural biological materials comprising highly ordered mineral crystals at high contents, *e.g.* about 40∼50 vol% in bone, ∼90 vol% in tooth enamel and ∼95 vol% in nacre^29^. Recently, learning from nature, a series of graphene-based artificial nacre have been fabricated^30^, in which the water-soluble derivative of graphene—graphene oxide (GO)—with many functional groups on the surface, were chemically cross-linked by polymer^31^,^32^,^33^,^34^,^35^, silk fibroin^36^, divalent ion (Mg^2+^, Ca^2+^)^37^, or borate^38^ and so on. These cross-linked highly concentrated GO layers resembled the “brick-and-mortar” nano-laminated architecture in nacre, alternatively packed with 95 vol% of aragonite calcium carbonate platelets and 5 vol% of protein layers, showing promising integrated mechanical properties. In metal matrix composites, although low graphene content does exhibit high reinforcing efficiency, the composites containing graphene at contents as high as that in biological materials have not been explored as they face tremendous challenges in fabrication.

Both theoretical^39^ and experimental^27^,^40^ results have proven the high reinforcing efficiency of graphene in metal matrix. Moreover, ordered graphene in matrix exhibited higher reinforcing efficiency. For example, as demonstrated in graphene-metal nanolayered composites, the strengths of Cu and Ni can be enhanced by 2.5 and 3.3 times by adding graphene of only ∼0.5 vol% and ∼0.34 vol% (*i.e.* single-atomic-layer graphene in metal layers with 70 and 100 nm repeated layer spacing), respectively^40^. Recently, simultaneous improvements on strength and toughness were achieved in our rGO/Cu^41^ and rGO/Al^42^ artificial nacres, in which the reinforcing efficiency of rGO was remarkably higher than that in the composites containing randomly dispersed rGO. In spite of great successes demonstrated by the metal matrix composites with graphene at low contents, composites with highly concentrated and ordered graphene are desired because: 1) biological materials is clearly inspiring for their ability to achieve novel properties by constructing such architecture with high content and ordered nanofiller; 2) ordering graphene implies higher reinforcing efficiency^43^; 3) high content graphene implies high density of interfaces for blocking dislocation motion being the dominant deformation mode in metals; 4) nanoconfinement effect benefited from high interface density makes it possible to transfer extraordinary properties of constituents at nanoscale (such as graphene and nano-metals in this work) to macroscale.

Herein, we present a strategy for fabricating bulk rGO-Cu nano-laminated composite with high rGO content (∼45 vol%) by bottom-up assembly and reduction of CuO/GO/CuO sandwich-like nanosheets. This process technique realized simultaneous uniform dispersion and ordering of high content graphene in metal matrix. Its microstructure and mechanical properties were characterized and discussed, and the as-obtained bulk nano-laminated composite exhibited recoverable deformation strain at least one order of magnitude greater than that of pure copper as well as increased specific strength because of high content graphene.

## Results and Discussion

### Preparation strategy

The challenges in fabricating graphene-metal bulk nano-laminated composite mainly lie in the following factors: homogenous dispersion and efficient ordering of graphene in metal matrix; consolidation of graphene-metal composite. These challenges will be much tougher when the graphene content is high. First, due to its large surface area and high surface energy that easily lead to agglomerations and restacking, as well as notable density difference between graphene and metals, it is extremely difficult to disperse graphene into metals homogeneously. Second, agglomeration and restacking of graphene also prevents consolidation of composite and results in a porous, fragile hybrid. Thirdly, no efficient method exists for odering graphene in metal matrix. Graphene could be incorporated into metal matrix in an ordered form by a modified powder metallurgy techonology^42^, by which graphene is absorbed onto the surface of flaky metal powder and then the composite powder is compacted into bulk, but consolidation will be a challenge when the fraction of graphene exceed a limit of about 3 vol%. The most frequent method for producing nanolayered composite is layer-by-layer assembly (LBL)^44^, which requires the methodical layering of the composite constituents via exposure of a substrate to alternating solutions of them. Although LBL can produce nanolayered composite with excellent properties, the low throughput limits this technique to film materials.

**Figure 1:**
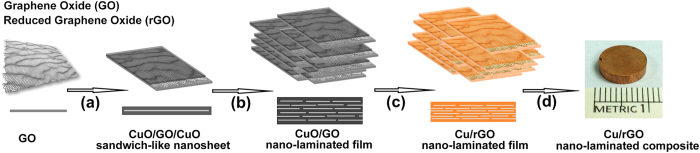
Schematic presentation for fabricating rGO-Cu nano-laminated composite by assembling sandwich-like units. (**a**) Deposition of CuO on both sides of graphene oxide (GO) to form CuO/GO/CuO sandwich-like nanosheet. (**b**) Assembling sandwich-like nanosheet via vacuum filtration; (**c**) Reducing CuO/GO in a H_2_/Ar mixed atmosphere. (**d**) Stacking the Cu/rGO film and then hot-pressing to obtain bulk nano-laminated composite, typically with a diameter of 10 mm.

In this work, we present a strategy to fabricate high content graphene-copper bulk nano-laminated composite by bottom-up assembly and reduction of CuO/GO/CuO sandwich-like nanosheets, by which the main obstacles mentioned above could be overcome to some extent. The whole fabrication process in this work was schematically shown in Fig. 1.

Pristine graphene is lack of enough active sites for anchoring and bonding metal ions and oxides, but GO is heavily oxygenated and is readily dispersed in water. GO can be synthesized from natural graphite flakes by a modified Hummers method. The GO played a role of template for fabricating CuO/GO/CuO composite powders. In order to assist the dispersion of GO in aqueous media and direct the deposition of CuO on the surface of GO, surfactant sodium dodecyl sulfate (SDS) was chosen to electrostatically adsorb and self-assemble onto the surface of GO. Cu cations were bound to the surfactant assembled onto the GO, forming CuO/GO/CuO sandwich-like nanosheets in alkaline solution with the decomposition of the added urea at elevated temperature. CuO was deposited on the both sides of the GO (Fig. 1a) and prevented them from restacking. Subsequently, the bottom-up assembly of CuO/GO/CuO sandwich-like nanosheets was carried out by vacuum filtering the parent solution (Fig. 1b), through which ordering GO in CuO was fast and efficiently realized. With further reducing the assembled CuO/GO/CuO films (Fig. 1c), individual dispersion of reduced graphene oxide (rGO) in Cu matrix at molecular level was achieved. Afterward, the reduced Cu/rGO/Cu films were stacked and consolidated by hot-pressing to produce bulk nano-laminated composites (Fig. 1d). Because the deposition of CuO on GO was carried out via a chemical route at molecular level and the remaining oxygen on rGO supplied strong bonding sites between rGO and Cu, strong interfacial strength in the final nanocomposites could be expected. Additionally, its consolidation behavior was similar to that of metal powders because it was realized *via* sintering surface Cu layer of the Cu/rGO/Cu sandwich-like nanosheets, by which composites with a high relative density could be achieved. In contrast, in traditional powder-metallurgy processes, strong interfacial bonding between graphene and metal matrix is by no means easy because they are merely blended. Moreover, sintering could not proceed easily because the metal powders are separated from each other by graphene with large surface area.

### Microstructure charaterazation

**Figure 2:**
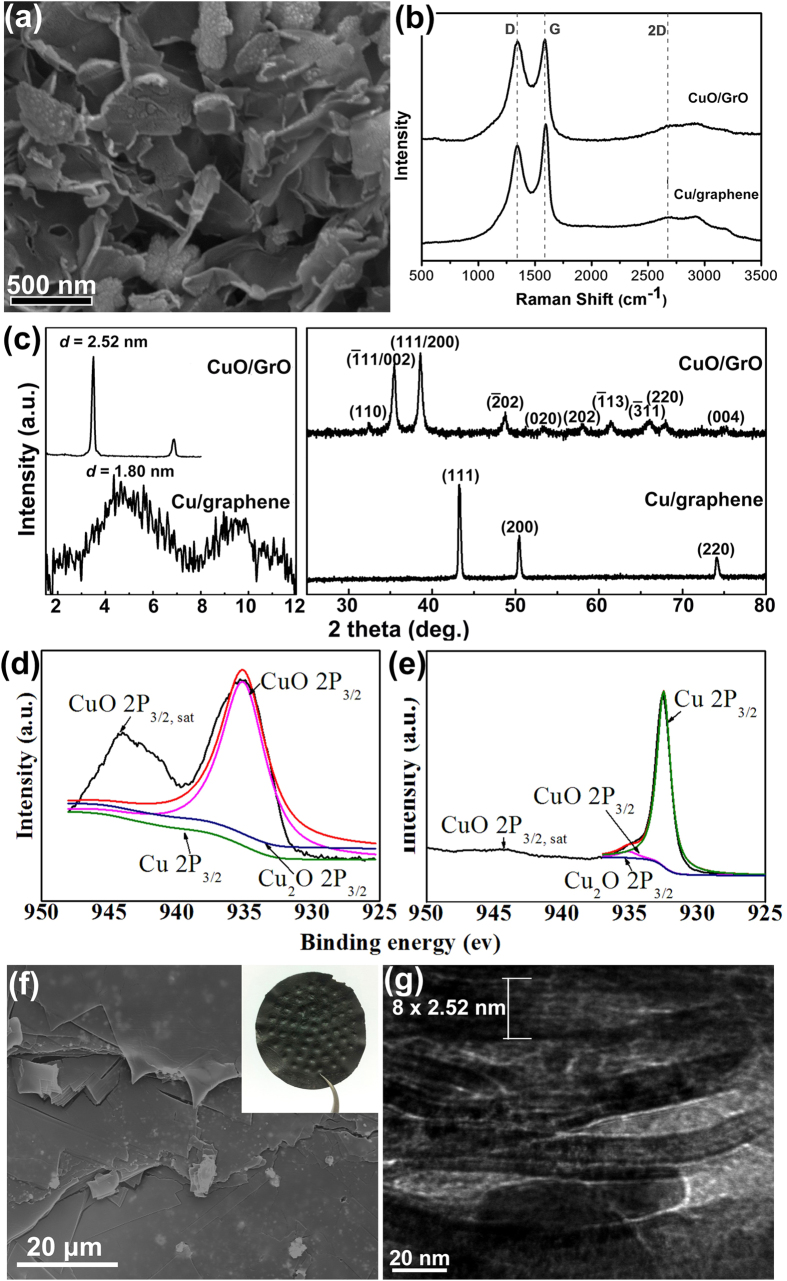
Structure characterization and phase analysis. (**a**) SEM image of dried powder of CuO/GO/CuO sandwich-like nanosheets. (**b**) Raman spectra and (**c**) small-angle and wide-angle XRD patterns of assembled films of CuO/GO and Cu/rGO composite. XPS spectra of Cu 2p for (**d**) CuO/GO/CuO sandwich-like nanosheet powder and (**e**) Cu/rGO composite. (**f**) SEM image of the fracture morphology and (**g**) TEM image of cross-section of assembled CuO/GO film show a lamellar structure, indicating an ordered arrangement of the CuO/GO/CuO sandwich-like nanosheets during vacuum filtering. The inset in (**d**) is the photo of assembled CuO/GO film with a diameter of 50 mm.

The morphology of dried CuO/GO/CuO sandwich-like nanosheets was characterized by scanning electron microscopy (SEM) and transmission electron microscopy (TEM) (Supplementary Figure S1). As shown in Fig. 2a, free-standing nanosheets with slightly curled edges are observed. The curled edge is common for a lamellar composite sheet after experiencing a dry process because of thermal mismatch between different constituents. Although the size of CuO/GO/CuO nanosheets could not be precisely measured in Fig. 2a, it is comparable to that of the starting GO sheets as shown in the AFM image (Supplementary Figure S2), ranging from hundreds of nanometers to several micrometers. No free CuO particles or naked GO sheets appear in SEM visualizations. We obtained Raman spectrum of the CuO/GO/CuO powder showing both D and G bands of GO structure with an *I*_D_/*I*_G_ band ratio of 0.98 (Fig. 2b). Wide-angle X-ray diffraction (WA-XRD) indicated no impurity.

Ordered assembly of the CuO/GO/CuO sandwich-like nanosheets *via* vacuum filtration was confirmed by small-angle X-ray diffraction (SA-XRD) (Fig. 2c), which indicated that the (001) *d*-spacing of the nano-laminated film is about 2.5 nm. This *d*-spacing obtained from XRD is consistent with that calculated from weight fraction and density of each constituent. According to the mole ratio of starting materials, the weight percentage of GO in the as-obtained CuO/GO composite was calculated to be ∼11 wt%, which is confirmed by thermal gravimetric analysis (Supplementary Figure S3). The thickness GO is measured to be ∼0.9 nm by atomic force microscope (AFM) (Supplementary Figure S2), which closes to that of a monolayer GO, and therefore the estimated thickness of CuO/GO/CuO sandwich-like nanosheets is comparable to the value of 2.5 nm obtained from XRD. The microstructures of the assembled film were characterized by SEM and TEM. SEM images (Fig. 2f, and Supplementary Figure S4) demonstrate the fracture morphology of the assembled film, and stepwise rupture of the laminated structure can be clearly discerned. The surface is smooth and no corrugation is observed, indicating the nanosheets are fully outspread at nanoscale. A typical TEM cross-sectional image of the assembled film shows a laminated structure (Fig. 2g), in which pores is observed because the assembled CuO/GO nano-laminated film was not completely densified during the self-assembly process (Supplementary Figure S4).

Because separated by CuO and Cu on surface, GO in the assembled CuO/GO nano-laminated film could be reduced without restacking, and rGO-Cu nano-laminated film was obtained finally. The WA-XRD (Fig. 2c) and X-ray photoelectron spectroscopy (XPS) (Fig. 2d,e) showed that CuO was completely reduced to Cu. As revealed by SA-XRD (Fig. 2c), nano-laminated structure is clearly discerned in the reduced film, although the degree of order is weaker than that before reduction. The (001) *d*-spacing of the nano-laminated structure was decreased to 1.8 nm because of reduction, and the fraction of rGO in Cu increased up to ∼13.5 wt. % correspondingly. Raman spectrum detected both D and G bands from rGO structure in the reduced film (Fig. 2b). The *I*_D_/*I*_G_ band ratio of 0.82 is smaller than that of assembled CuO/GO film, indicating fewer defects in the rGO. Cu/rGO nano-laminated films were then stacked and hot-pressed to prepare bulk Cu/rGO nano-laminated composite. The density of the bulk composite was measured to be 6.1 g cm^−3^ by the Archimedes method. This value is much smaller than that of pure copper (8.9 g cm^−3^), but the bulk composite is near fully densified in view of high content rGO in the composite. The high density meets our anticipation as the consolidation that realized through the Cu surface layer of Cu/rGO/Cu sandwich-like nanosheets was similar with that of pure metal.

**Figure 3:**
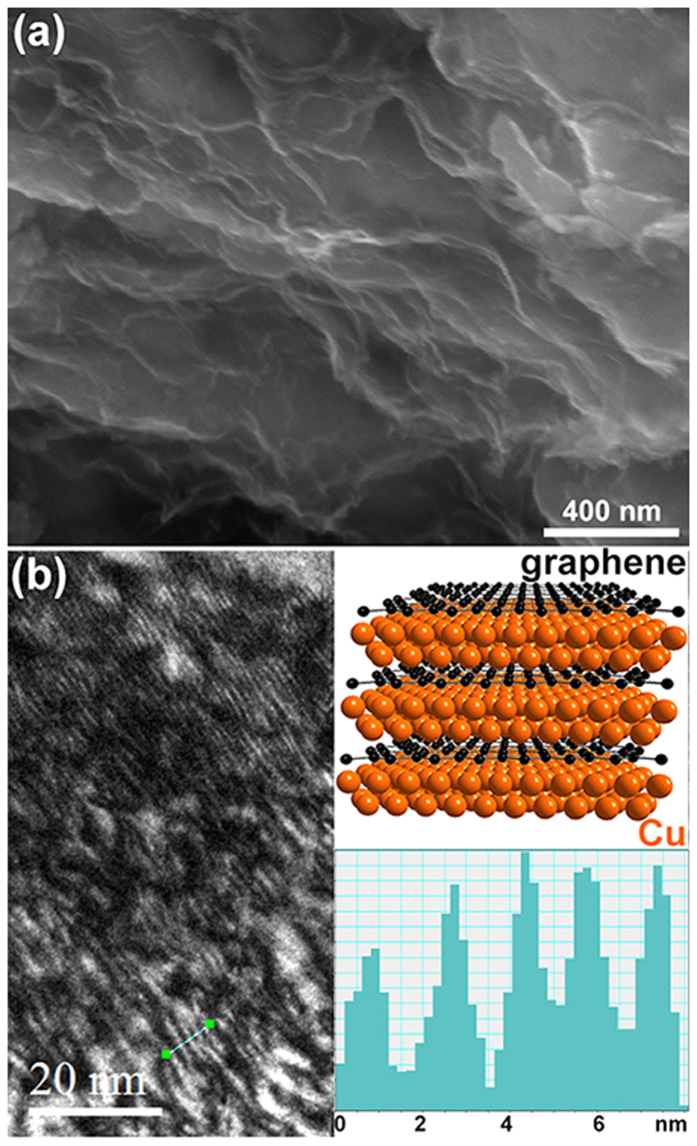
Microstructure characterization for the bulk composite. (**a**) SEM image of fracture surface of Cu/rGO bulk nano-laminated composite. (**b**) TEM image shows nano-laminated microstructure in the composite. The upper inset shows a model of the microstructure, and the lower inset is the gray scale section analysis for the lamellae as marked by the short bar directly labeled on the image.

Nano-laminated structure was finally obtained in the bulk Cu/rGO composites. SEM image (Fig. 3a) demonstrates the fracture morphology of the bulk Cu/rGO nano-laminated composite. The fracture morphology is obviously different with pure copper that typically shows well-developed dimples over the entire fractured surface. The rGO are parallel to others neighbor sheets and their edges are staggered, which is reminiscent of a nacre-like structure^30^. TEM reveals well defined and highly ordered self-assemblies, showing a lamellar structure consisting of alternating sbu-2 nm Cu and rGO. The periodicity in the lamellar structure is about 1.7 nm (Fig. 3b), which closes to the value obtained from SA-XRD for the reduced nano-laminated film (Fig. 2c).

### Mechanical properties

**Figure 4:**
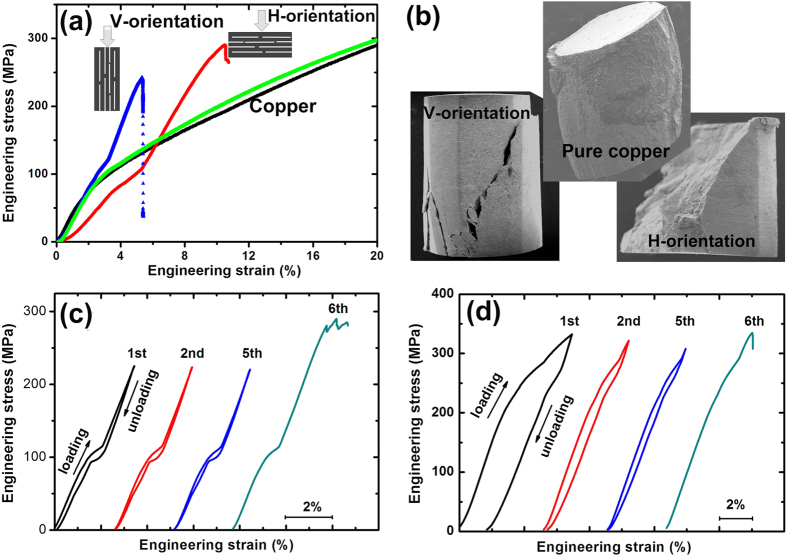
Mechanical properties of the bulk composite. (**a**) Compressive strain-stress curves for the pure Cu (two directions), and the V- and H-orientation of the composite. (**b**) SEM images of the fractured specimens after compressive testing for the pure Cu, and the V- and H-orientation of the composite. The cyclic compressive strain-stress curves for (**c**) the V-orientation and (**d**) the H-orientation.

The mechanical properties along the directions both parallel (V-) and perpendicular (H-) to lamellae were investigated using compressive testing. Typical stress–strain curves of the composites are shown in Fig. 4a. The compressive ultimate strength (at the point of shear failure, as shown in Fig. 4b) for the V- and H-orientations was 246 ± 20 MPa and 294 ± 12 MPa, respectively, which is comparable to that of monolithic pure Cu. It is worth mentioning here that the density of the composite is only ∼69% of that of the Cu matrix because of high content rGO, indicating that the specific strength is significantly enhanced because of rGO reinforcing. More interesting, the as-obtained nano-laminated composite show very high recoverable deformation ability, which is at least one order of magnitude greater than that of pure Cu (with <0.5% elastic strain), and the ability of the H-orientation is higher than that of the V-orientation (Fig. 4a). The recoverable deformation abilities of the as-obtained nano-laminated composites were supported by the cyclic compressive strain-stress curves for both the V-orientation (Fig. 4c) and the H-orientation (Fig. 4d). Exceeding the recoverable deformation ability, the failure of the composite follows a shear mode for both V- and H-orientations, while the pure Cu without the presence of rGO shows plastic deformation (Fig. 4b).

The large recoverable deformation strain of the bulk Cu/rGO composite investigated in this work could be attributed to a combined effect: nanoconfinement effect induced by high content rGO and thus high interface density, reinforcing effect of rGO, as well as nano-laminated architecture. While bulk metals have high strength but limited ability to deform elastically, nanoscale metals could exhibit high elastic strain and even rubber-like behavior^45^,^46^. By creating nanocomposites, the exceptional inherent mechanical properties of low-dimensional constituents could be translated into bulk materials because of synergistic and nanoconfinement effects^47^,^48^. In the composite investigated, the high interface density induced by high content rGO sheets confine the Cu matrix in two-dimensional spaces with a thickness of sub-2 nm (Fig. 3b), and prevent the nanoscale Cu matrix from growing up during fabrication process and compression, transferring and maintaining mechanical properties of the copper at nanoscale to macroscale. On the other hand, highly concentrated graphene will also show unique collective feature as compared with single-atomic graphene layer that is highly rigid in plane and flexible out of plane. For example, nanoarchitectured materials composed of fullerene-like spheroids and disordered graphene layers showed largely linear and recoverable up to unusually high strains (*ca* 6%)^49^. Therefore, a possible deformation scenario could be depicted based on the synergic effect between confined Cu and highly concentrated and ordered rGO. Upon applying force, the copper become compressed and the rGO kinked at first. Subsequently, shear force formed with further increasing applied force, and this turning point could correspond to the inflexions in the strain-stress curves observed for both the V- and H-orientations. The stiffness of the H-orientation is obviously lower than that of the V-orientation (Fig. 4a), which is consistent with other layered nanocomposites consisting of “hard” and “soft” constituents^50^. Because of high volume fraction (as high as ∼45 vol%) of rGO and Cu lamella with thickness as thin as ∼1 nm, inside which dislocation formation and motion being the dominant deformation mode in metals became very difficult, the composite exhibited a brittle fracture behavior when the strain exceeded the recoverable deformation ability. Therefore, the failure stress was dominated by the interfacial bonding strength in the Cu/rGO nano-laminated composites. Because of similar wet chemical route with the molecular-level mixing process, the interfacial bonding between rGO and Cu in the final nanocomposites could be strong because it is mediated by the residual oxygen^27^, and thus the Cu/rGO composite showed both increased specific failure strength and large recoverable deformation strain as compared to the Cu sample without the presence of rGO. The decreased failure strain could be improved by increasing the thickness of Cu lamella to enhance the ability of dislocation formation and motion.

## Conclusions

In summary, inspired by nature, we successfully fabricated a high content rGO (∼45 vol%) reinforced Cu bulk nano-laminated composite. The molecular level dispersing and ordering high content rGO were simultaneously realized by assembling and reducing CuO/GO/CuO sandwich-like nanosheets. This process is also expected to be applied to GO-ceramic nanocomposites by omitting the reduction step. Therefore, the unique strategy will greatly expand the application range of graphene or its derivates as a promising reinforcement in both metal and ceramic matrixes with a wide range of content. Thanks to the high content rGO, high interfacial density, as well as nano-laminated structure, the as-obtained nano-laminated bulk composite shows increased specific strength and at least one order of magnitude greater recoverable deformation ability as compared with the pure Cu. Combined with its high mechainical strength, such composites would be attractive as shock absorbers and energy storage devices.

## Methods

### Synthesis of CuO/GO/CuO Sandwich-like Nanosheets

In a typical procedure, 200 mg graphene oxide (GO, prepared by the Hummer’s method) was dispersed into 200 mL deionized water via ultrasonic under 500 W for 2 h, forming a GO aqueous dispersion with a concentration of 1 mg/mL, and then mixed with 75 mL of sodium dodecyl sulfate solution (0.5 M). The mixture was sonicated for 15 min. Then 100 mL of 0.2 M Cu(NO_3_)_2_•3H_2_O solution was added dropwise under vigorous stirring followed by the addition of 400 mL of 0.25 M urea solution and 300 mL of deionized water. The resulting mixture was further stirred in a sealed glass flask at 90 °C for 16 h, and CuO/GO/CuO nanosheet solution was obtained after the product was cooled to room temperature.

### Fabrication of CuO/GO/CuO film

GO/CuO multilayered composite films were prepared by vacuum filtering the resulting CuO/GO/CuO solution, and were peeled off from the funnel after a brief air-drying period. The films were finally dried in an air dry oven at 60 °C for 6 h for the next step.

### Fabrication of bulk graphene-Cu nano-laminated composite

The dried GO/CuO multilayered composite films was treated in 20% H_2_/Ar gas at 450 °C for 2 h for reducing CuO and GO as well as decomposing residual organic molecular. The as-obtained Cu/rGO multilayered composite films were further stacked and consolidated by hot-pressing at 900 °C for 20 min in a vacuum of 0.01 Pa with an applied pressure of 50 MPa. The final size of the bulk Cu/rGO nano-laminated composite was 10 mm in diameter and 3.5 mm in thickness typically.

### Characterization

The morphology and structure of GO were characterized by AFM (‘E-Sweep’ of the NanoNavi-Series) and Raman spectroscopy (Senterra R200-L, Bruker). Phase and elemental analyses were performed by XRD (D8 Advance, Bruker) and XPS (AXIS Ultra DLD, Shimadzu-Kratos). The microstructures of the film and composite were observed by SEM (FEI SIRION 200) and TEM (JEOL JEM-2100F). For compressive testing, the obtained samples were cut using a wire electrical discharging machine along the direction both parallel and perpendicular to the layered direction and were polished to cylinder shape with a height of 3.5 mm and a diameter of 2 mm. The compressive testing was conducted using a servo-hydraulic materials testing system (Zwick/Roell Z020) at room temperature at a strain rate of 1 × 10^−4^ s^−1^.

## Additional Information

**How to cite this article**: Xiong, D.-B. *et al.* High content reduced graphene oxide reinforced copper with a bioinspired nano-laminated structure and large recoverable deformation ability. *Sci. Rep.* **6**, 33801; doi: 10.1038/srep33801 (2016).

## Supplementary Material

Supplementary Information

## References

[b1] Meyers M. A., McKittrick J. & Chen P.-Y. Structural biological materials: critical mechanics-materials connections. Science 339, 773–779 (2013). 23413348 10.1126/science.1220854

[b2] Wegst U. G. K., Bai H., Saiz E., Tomsia A. P. & Ritchie R. O. Bioinspired structural materials. Nat. Mater. 14, 23–36 (2015). 25344782 10.1038/nmat4089

[b3] Studart A. R. Towards high-performance bioinspired composites. Adv. Mater. 24, 5024–5044 (2012). 22791358 10.1002/adma.201201471

[b4] Dunlop J. W. C. & Fratzl P. Biological composites. Annu. Rev. Mater. Res. 40, 1–24 (2010).

[b5] Schmidt D. F. Nanolaminates – bioinspired and beyond. Mater. Lett. 108, 328–335 (2013).

[b6] Dunlop J. W. C. & Fratzl P. Multilevel architectures in natural materials. Scrip. Mater. 68, 8–12 (2013).

[b7] Ritchie R. O. The conflicts between strength and toughness. Nat. Mater. 10, 817–822 (2011). 22020005 10.1038/nmat3115

[b8] Wang J. F., Chen Q. F. & Tang Z. Y. Layered nanocomposites inspired by the structure and mechanical properties of nacre. Chem. Soc. Rev. 41, 1111–1129 (2012). 21959863 10.1039/c1cs15106a

[b9] Yao H. B., Ge J., Mao L. B., Yan Y. X. & Yu S. H. 25^th^ anniversary article: artificial carbonate nanocrystals and layered structural nanocomposites inspired by nacre: synthesis, fabrication and applications. Adv. Mater. 26, 163–188 (2014). 24338814 10.1002/adma.201303470

[b10] Tang Z. Y., Kotov N. A., Magonov S. & Ozturk B. Nanostructured artificial nacre. Nat. Mater. 2, 413–418 (2003). 12764359 10.1038/nmat906

[b11] Munch E. *et al.* Tough, bio-inspired hybrid materials. Science 322, 516–520 (2008). 19056979 10.1126/science.1164865

[b12] Dwivedi G., Flynn K., Resnick M., Sampath S. & Gouldstone A. Bioinspired hybrid materials from spray-formed ceramic templates. Adv. Mater. 27, 3073–3078 (2015). 25855576 10.1002/adma.201500303

[b13] Bouville F. *et al.* Strong, tough and stiff bioinspired ceramics from brittle constituents. Nat. Mater. 13, 508–514 (2014). 24658117 10.1038/nmat3915

[b14] Podsiadlo P. *et al.* Ultrastrong and stiff layered polymer nanocomposites. Science 318, 80–83 (2007). 17916728 10.1126/science.1143176

[b15] Bonderer L. J., Studart A. R. & Gauckler L. J. Bioinspired design and assembly of platelet reinforced polymer films. Science, 319, 1069–1073 (2008). 18292337 10.1126/science.1148726

[b16] Geim A. K. Graphene: status and prospects. Science 324, 1530–1534 (2009). 19541989 10.1126/science.1158877

[b17] Qiu L., Liu J. Z., Chang S. L. Y., Wu Y. Z. & Li D. Biomimetic superelastic graphene-based cellular monoliths. Nat. Comm. 3, 1241 (2012). 10.1038/ncomms2251 23212370

[b18] Chen Z. P. *et al.* Three-dimensional flexible and conductive interconnected graphene networks grown by chemical vapor deposition. Nat. Mater. 10, 424–428 (2011). 21478883 10.1038/nmat3001

[b19] Sun H. Y., Xu Z. & Gao C. Multifunctional, ultra-flyweight, synergistically assembled carbon aerogels. Adv. Mater. 25, 2554–2560 (2013). 23418099 10.1002/adma.201204576

[b20] Hu H., Zhao Z. B., Wan W. B., Gogotsi Y. & Qiu J. S. Ultralight and highly compressible graphene aerogels. Adv. Mater. 25, 2219–2223 (2013). 23418081 10.1002/adma.201204530

[b21] Xu Z. & Gao C. Graphene chiral liquid crystals and macroscopic assembled fibres. Nat. Comm. 2, 571 (2011). 10.1038/ncomms1583 PMC3247827 22146390

[b22] Dikin D. A. *et al.* Preparation and characterization of graphene oxide paper. Nature 448, 457–460 (2007). 17653188 10.1038/nature06016

[b23] Huang X., Qin X. Y., Boey F. & Zhang H. Graphene-based composites. Chem. Soc. Rev. 41, 666–686 (2012). 21796314 10.1039/c1cs15078b

[b24] Potts J. R., Dreyer D. R., Bielawski C. W. & Ruoff R. S. Graphene-based polymer nanocomposites. Polymer 52, 5–25 (2011).

[b25] Novoselov K. S. *et al.* A roadmap for graphene. Nature 490, 192–200 (2012). 23060189 10.1038/nature11458

[b26] Walker L. S., Marotto V. R., Rafiee M. A., Koratkar N. & Corral E. L. Toughening in graphene ceramic composites. ACS Nano 5, 3182–3190 (2011). 21443241 10.1021/nn200319d

[b27] Hwang J. *et al.* Enhanced mechanical properties of graphene/copper nanocomposites using a molecular-level mixing process. Adv. Mater. 25, 6724–6729 (2013). 23983045 10.1002/adma.201302495

[b28] Wang J. Y. *et al.* Reinforcement with graphene nanosheets in aluminum matrix composites. Scrip. Mater. 66, 594–597 (2012).

[b29] Ji B. H. & Gao H. J. Mechanical properties of nanostructure of biological materials. J. Mech. Phys. Solids 52, 1963–1990 (2004).

[b30] Cheng Q. F., Duan J. L., Zhang Q. & Jiang L. Learning from nature: constructing integrated graphene-based artificial nacre. ACS Nano 9, 2231–2234 (2015). 25763684 10.1021/acsnano.5b01126

[b31] Li Y.-Q., Yu T., Yang T.-Y., Zheng L.-X. & Liao K. Bio-inspired nacre-like composite films based on graphene with superior mechanical, electrical and biocompatible properties. Adv. Mater. 24, 3426–3431 (2012). 22730223 10.1002/adma.201200452

[b32] Park Y. S., Dikin D. A., Nguyen S. T. & Ruoff R. S. Graphene oxide sheets chemically cross-linked by polyallylamine. J. Phys. Chem. C 113, 15801–15804 (2009).

[b33] Zhao X. L., Xu Z., Zheng B. N. & Gao C. Macroscopic assembled, ultrastrong and H_2_SO_4_-resitant fibres of polymer-grafted graphene oxide. Sci. Rep. 3, 3164 (2013). 24196491 10.1038/srep03164 PMC3819614

[b34] Putz K. W., Compton O. C., Palmeri M. J., Nguyen S. T. & Brinson L. C. High-nanofiller-content graphene oxide-polymer nanocomposites *via* vacuum-assisted self-assembly Adv. Funct. Mater. 20, 3322–3329 (2010).

[b35] Huang L., Li C., Yuan W. J. & Shi G. Q. Strong composite films with layered structures prepared by casting silk fibroin-graphene oxide hydrogels. Nanoscale 5, 3780–3786 (2013). 23538717 10.1039/c3nr00196b

[b36] Zhang M., Huang L., Chen J., Li C. & Shi G. Q. Ultratough, ultrastrong, and highly conductive graphene films with arbitrary sizes. Adv. Mater. 26, 7588–7592 (2014). 25250891 10.1002/adma.201403322

[b37] Park S. *et al.* Gaphene oxide papers modified by divalent ions-enhancing mechanical properties *via* chemical cross-linking. ACS Nano 2, 572–578 (2008). 19206584 10.1021/nn700349a

[b38] An Z., Compton O. C., Putz K. W., Brinson L. C. & Nguyen S. T. Bio-inspired borate cross-linking in ultra-stiff graphene oxide thin films. Adv. Mater. 23, 3842–3846 (2011). 21793051 10.1002/adma.201101544

[b39] Chang S.-W., Nair A. K. & Buehler M. J. Nanoindentation study of size effects in nickel-graphene nanocomposites. Phil. Mag. Lett. 93, 196–203 (2013).

[b40] Kim Y. *et al.* Strengthening effect of single-atomic-layer graphene in metal-graphene nanolayered composites. Nature Comm. 4, 2114 (2013). 10.1038/ncomms3114 23820590

[b41] Xiong D.-B. *et al.* Graphene-and-copper artificial nacre fabricated by a preform impregnation process: bioinspired strategy for strengthening-toughening of metal matrix composite. ACS Nano 9, 6934–6943 (2015). 26083407 10.1021/acsnano.5b01067

[b42] Li Z. *et al.* Enhanced mechanical properties of graphene (reduced graphene oxide)/aluminum composites with a bioinspired nanolaminated structure. Nano Lett. 15, 8077–8083 (2015). 26574873 10.1021/acs.nanolett.5b03492

[b43] Greil P. Perspectives of nano-carbon based engineering materials. Adv. Eng. Mater. 17, 124–137 (2015).

[b44] Richardson J. J., Björnmalm M. & Caruso F. Technology-driven layer-by-layer assembly of nanofilms. Science 348, aaa2491 (2015). 10.1126/science.aaa2491 25908826

[b45] Yue Y. H. *et al.* Crystalline liquid and rubber-like behavior in Cu nanowires. Nano Lett. 13, 3812–3816 (2013). 23898785 10.1021/nl401829e

[b46] Yue Y. H., Liu P., Zhang Z., Han X. D. & Ma E. Approaching the theoretical elastic strain limit in copper nanowires. Nano Lett. 11, 3151–3155 (2011). 21766832 10.1021/nl201233u

[b47] Hao S. J. *et al.* A transforming metal nanocomposite with large elastic strain, low modulus, and high strength. Science 339, 1191–1194 (2013). 23471404 10.1126/science.1228602

[b48] Zhang J. S. *et al.* A biopolymer-like metal enabled hybrid material with exceptional mechanical prowess. Sci. Rep. 5, 8657 (2015). 25665501 10.1038/srep08357 PMC4322361

[b49] Zhao Z. S. *et al.* Nanoarchitectured materials composed of fullerene-like spheroids and disordered graphene layers with tunable mechanical properties. Nat. Comm. 6, 6212 (2015). 10.1038/ncomms7212 25648723

[b50] Lee J.-H. *et al.* High strain rate deformation of layered nanocomposites. Nat. Comm. 3, 1164 (2012). 10.1038/ncomms2166 23132014

