# Supplementary material for: High content reduced graphene oxide reinforced copper with a bioinspired nano-laminated structure and large recoverable deformation ability
Source: Sci Rep. 2016 Sep 20;6:33801. doi: 10.1038/srep33801 (PMC5029288; doi:10.1038/srep33801)
Supplement: Supplementary Information [file srep33801-s1.pdf]

Supplementary Information

High content reduced graphene oxide reinforced copper with a bioinspired nano-laminated structure and large recoverable deformation ability

Ding-Bang Xiong, Mu Cao, Qiang Guo, Zhanqiu Tan, Genlian Fan, Zhiqiang Li*, and Di Zhang*

State Key Laboratory of Metal Matrix Composites, Shanghai Jiao Tong University, Shanghai, 200240, China.
*lizhq@sjtu.edu.cn; zhangdi@sjtu.edu.cn.

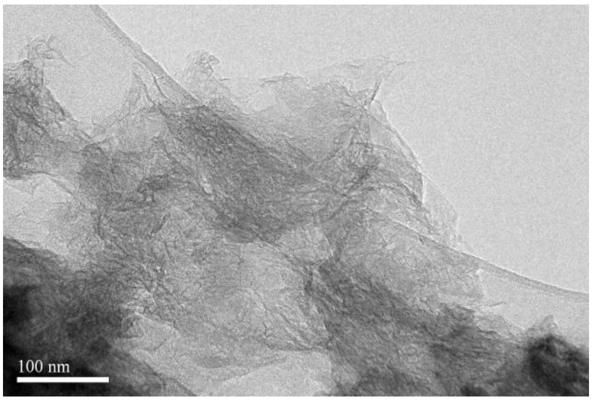

Figure S1. TEM image of the CuO/GO/CuO sandwich-like nanosheets.

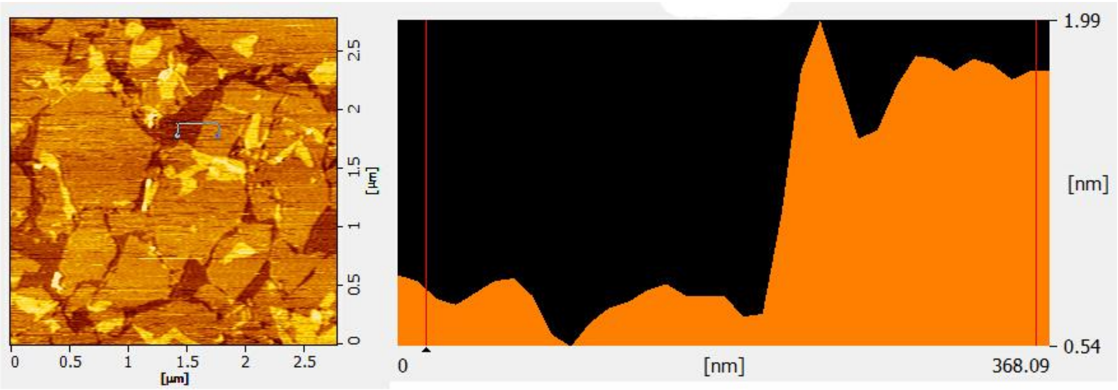

Figure S2. AFM analysis for the morphology of the GO obtained by the Hummer's method, indicating the thickness of ~0.9 nm and the sizes ranging from hundreds of nanometers to several micrometers.

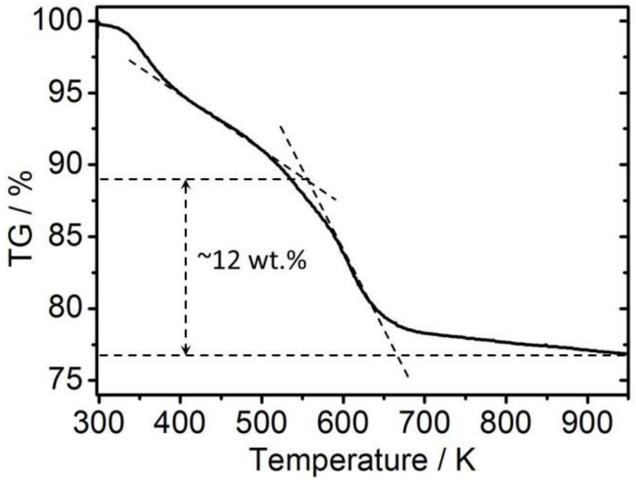

Figure S3. TG analysis for the CuO/GO/CuO sandwich-like nanosheets.

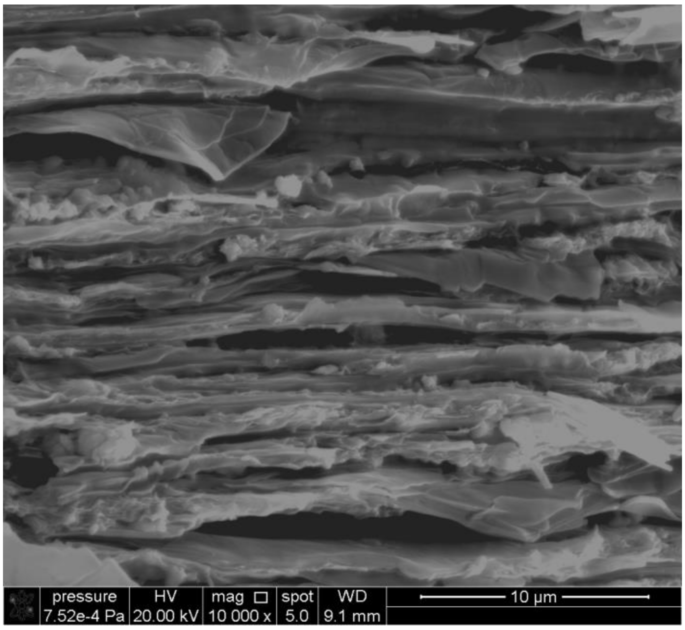

Figure S4. SEM image for the cross section of the assembled GO/CuO film.
